# Abnormal H3K27me3 underlies degenerative spermatogonial stem cells in cryptorchid testis

**DOI:** 10.1242/dev.204239

**Published:** 2025-01-16

**Authors:** Kazushige Kuroha, Ivana Dočkal, Uroš Radović, Kuniko Nakajima, Ikue Hoshi, Shion Matsuda, Noriko Kojitani, Kazuyuki Ohbo, Shin-ichi Tomizawa

**Affiliations:** Department of Histology and Cell Biology, Yokohama City University School of Medicine, Yokohama 236-0004, Japan

**Keywords:** Cryptorchidism, Spermatogenesis, Spermatogonial stem cells, Epigenetics, Histone modification

## Abstract

Cryptorchidism is the most frequent congenital defect in newborn males characterized by the absence of the testis from the scrotum. Approximately 90% of individuals with untreated bilateral cryptorchidism exhibit azoospermia due to defective spermatogenesis in the affected testis. Although abnormal spermatogonial stem cell maintenance or differentiation is suggested to cause germ cell degeneration in the cryptorchid testis, the underlying molecular mechanisms remain unclear. Here, we profiled spermatogonial epigenetic landscapes using surgically induced cryptorchid testis in the mouse. We show that cryptorchidism leads to alterations in local, but not global, H3K27me3 and H3K9me3 in undifferentiated spermatogonia. Of these, the loss of H3K27me3 was correlated with activation of developmental and proapoptotic pathway genes that are repressed by the polycomb machinery in germ cells. Cryptorchid spermatogonia exhibit an increase of the H3K27me3 demethylases KDM6A and KMD6B. Furthermore, we reveal that an increased temperature leads to *Kdm6a/b* upregulation in germline stem cells cultured *in vitro*. Thus, our study suggests that temperature-dependent histone demethylation may induce mRNA dysregulation due to the partial loss of H3K27me3 in spermatogonia.

## Teaser

**Summary:** Partial loss of H3K27me3 correlated with developmental and proapoptotic gene upregulation underlies abnormal spermatogonial stem cell maintenance and differentiation in cryptorchid testis in mice.

## INTRODUCTION

Cryptorchidism is a condition characterized by the undescended testis into the scrotum and is one of the most frequent congenital anomalies in newborn males affecting 1-9% of boys in some North American and European countries (Braga and Lorenzo, 2017; Hadziselimovic, 2017; Holmboe, 2024). Cryptorchid testis represents deficient germ cell development and increased incidence of testicular tumors, and the bilateral form of cryptorchidism poses a risk factor for infertility (Cobellis et al., 2014). The pathology of cryptorchidism has been studied in animals using artificial cryptorchid testis induced by surgery. The treated testes exhibit variable effects on spermatogenesis: in the severest cases, it could leave only undifferentiated spermatogonia and Sertoli cells remaining in the seminiferous tubules, and other types of germ cells are lost (Farooqui et al., 1997, 2001; Kazusa et al., 2004; Nishimune et al., 1978; Zhang et al., 2002). The remaining spermatogonial stem cells (SSCs) among undifferentiated spermatogonia also represent reduced stem cell activity and alterations in marker gene expression (Ferguson et al., 2013; Kamisawa et al., 2012). Thus, abnormal SSC characteristics and differentiation capability may be crucial causative factors of degenerative germ cells in the cryptorchid testis.

Mammalian spermatogenesis is a temperature-sensitive process that proceeds in scrotum, which is 2-7°C lower than the abdominal cavity where the cryptorchid testis is positioned (Moore and Quick, 1924). Even a 15-min exposure of scrota containing testis to heat induces apoptosis of germ cells in the testis (Lue et al., 1999; Miura et al., 2002). Meiotic progression is inhibited at elevated temperatures in testis explants (Hirano et al., 2022). Furthermore, *in vitro* studies have shown that temperature affects spermatogonial DNA synthesis, SSC self-renewal, and SSC differentiation (Gao et al., 2023; Nakamura et al., 1987; Wang et al., 2019). Thus, high temperatures affect various types of germ cells in different ways. While inhibition of normal hormonal regulation is one possible cause, these reports support the hypothesis that increased temperature of the undescended testis is a crucial aspect that contributes to defective spermatogenesis (Cobellis et al., 2014; Mieusset and Bujan, 1995). However, underlying molecular mechanisms linking temperature and spermatogenesis remain poorly understood.

Male germ cell development is a complex process associated with dynamic gene expression precisely controlled by epigenetic mechanisms. In particular, reports suggest the importance of the SSC epigenome for spermatogenesis. During SSC self-renewal or differentiation, the *de novo* DNA methyltransferases DNMT3A and DNMA3B, as well as histone modifiers such as KMT2B, KDM1A, and DOT1L, play crucial roles (Dura et al., 2022; Lambrot et al., 2015; Lin et al., 2022; Shirakawa et al., 2013; Tomizawa et al., 2018). SSC self-renewal also requires polycomb repressive complex 1 (PRC1), which catalyzes histone H2A lysine 119 ubiquitylation (H2AK119Ub) as well as polycomb repressive complex 2 (PRC2), which catalyzes histone H3 lysine 27 tri-methylation (H3K27me3) (Hu et al., 2024; Maezawa et al., 2017). Moreover, SETDB1 and SUV39H proteins required for H3K9me2/3 are also crucial for SSC maintenance or progression of spermatogenesis (An et al., 2014; Liu et al., 2014; Peters et al., 2001). Thus, SSC epigenome integrity is central for the sustained maintenance and differentiation of germ cells in the testis.

Previous studies have shown that epigenetic mechanisms respond to temperature changes. Temperature-dependent sex determination in different species is correlated with DNA methylation and histone modification changes (Matsumoto et al., 2013, 2016; Navarro-Martín et al., 2011; Parrott et al., 2014; Radhakrishnan et al., 2017; Shao et al., 2014). Expression of *Drosophila* Polycomb group (PcG) genes responsible for the repressive H3K27me3 modification have also been shown to be temperature sensitive (Voigt and Froschauer, 2023). Moreover, one of the H3K27 demethylases, KDM6B (also known as JMJD3), promotes male sex determination only at low temperatures in a turtle species (Ge et al., 2018). Additionally, another repressive mark, H3K9me3, is altered and causes endogenous repeat de-repression in *Caenorhabditis elegans* exposed to high temperatures (Klosin et al., 2017). Despite these findings, it remains unexplored whether epigenetic integrity is properly maintained in the cryptorchid testis exposed to high temperatures.

In this study, we profiled the histone modification status of undifferentiated spermatogonia using surgically induced cryptorchid testis. We focus on two modifications crucial for SSCs: H3K27me3 and H3K9me3. Our histological observations demonstrated small changes in H3K27me3 and H3K9me3 levels, but chromatin immunoprecipitation sequencing (ChIP-seq) revealed alterations of a subset of local H3K27me3 and H3K9me3 peaks. While the affected H3K9me3 peaks were not correlated with transcriptional changes, H3K27me3 loss was correlated with mRNA upregulation of associated genes. In particular, developmental genes and apoptosis genes that are normally under H3K27me3 repression showed activation in cryptorchid spermatogonia. Further investigation showed that H3K27me3 demethylases KDM6A and KDM6B are upregulated in cryptorchid spermatogonia. We also show evidence that *Kdm6a/b* mRNA is upregulated in germline stem cells (GSCs) cultured under a high-temperature condition. These results suggest the possibility that epigenome abnormalities in cryptorchid spermatogonia induce premature developmental gene expression that may interfere with normal spermatogenesis.

## RESULTS

### Cryptorchidism affects spermatogonial maintenance

**Fig. 1.**
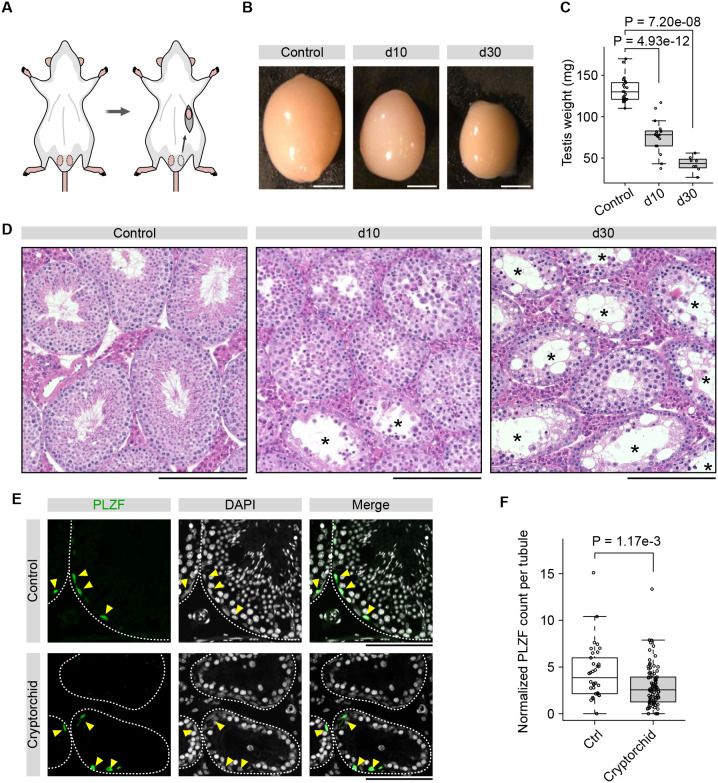
**Cryptorchidism affects spermatogonial maintenance and differentiation.** (A) Scheme showing cryptorchid surgery. One testis is placed in the abdominal cavity to induce cryptorchidism. (B) Representative images showing control and cryptorchid testis at d10 and d30 after surgery. Scale bars: 2 mm. (C) Weight of the testis in control (*n*=25), d10 cryptorchid (*n*=18), and d30 cryptorchid (*n*=8) samples. (D) H&E staining for testicular sections showing control, d10 cryptorchid, and d30 cryptorchid samples. Asterisks indicate degenerated tubules showing substantial loss of germ cells. Scale bars: 200 μm. (E) IF images showing control and cryptorchid (d30) spermatogonia stained with antibodies for PLZF (green). Gray, DAPI. Yellow arrowheads indicate PLZF^+^ spermatogonia. Dashed lines delineate seminiferous tubules. Scale bars: 100 μm. (F) Quantification of PLZF^+^ spermatogonia from IF images normalized for the seminiferous tubule diameter. *n*=34 (control) and 84 (cryptorchid) seminiferous tubules. (C,F) *P*-values are indicated above the plots (one-tailed Wilcoxon rank sum test). In box plots, data are median (horizontal line), 25th and 75th percentile; whiskers show the range; dots represent data points.

To systematically investigate how cryptorchidism affects spermatogonial maintenance, we performed surgery to induce unilateral cryptorchidism in mice by attaching one of the testes to the abdominal wall according to a method reported previously (Zheng et al., 2019) (Fig. 1A). We collected testes at 10 and 30 days after surgery (d10 and d30) and used these for the downstream analysis. Because the effect of cryptorchid surgery on germ cell degeneration can be inconsistent, likely due to the differing positions of the testis inside the abdominal cavity (Hirano et al., 2022), we evaluated the size and histology of our samples. Our comparison showed that cryptorchid testes were consistently smaller than untreated control testes (Fig. 1B), and the weights were significantly reduced (Fig. 1C). Hematoxylin and Eosin (H&E) staining of testicular sections confirmed time-dependent degeneration of seminiferous tubules in the cryptorchid testes with increased numbers of tubules showing a more severe loss of germ cells at d30 than d10 (Fig. 1D). Consistent with previous reports (Cai et al., 2021; Clegg, 1963; Liu et al., 2012; Zheng et al., 2019), we observed large vacuoles and abnormal morphology of germ cells as well as shrinkage of seminiferous tubules of treated sections (Fig. 1D).

To investigate whether spermatogonial maintenance is affected in the cryptorchid testis, we performed immunofluorescence (IF) for PLZF (ZBTB16), a spermatogonial marker expressed in undifferentiated spermatogonia and differentiation-committed progenitor spermatogonia (Hermann et al., 2009, 2010; Phillips et al., 2010). Despite the substantial effect on germ cells in cryptorchid testis, we observed the presence of PLZF^+^ spermatogonia near the basement membrane in both control and cryptorchid sections (Fig. 1E). However, quantification of cell numbers normalized for the tubule diameter showed a significant decrease of PLZF^+^ spermatogonia per seminiferous tubule in cryptorchid testis. This result suggests that spermatogonial maintenance is affected in cryptorchid testis (Fig. 1E,F).

### Maintenance of global histone modification status in cryptorchid spermatogonia

**Fig. 2.**
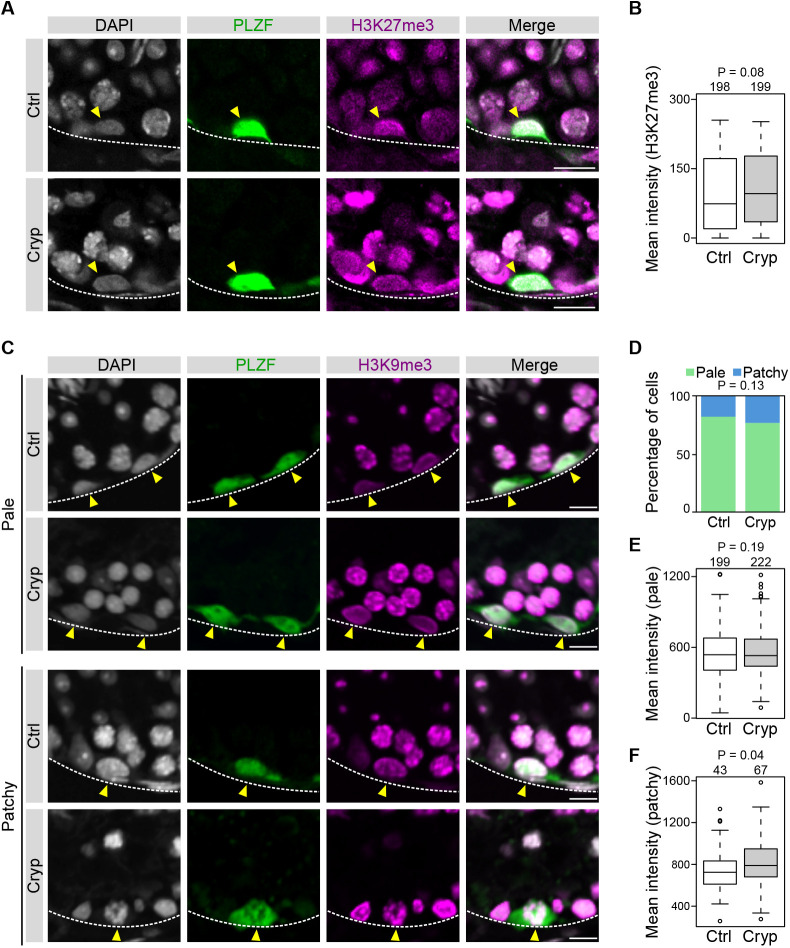
**Global levels of H3K27me3 and H3K9me3 in cryptorchid spermatogonia.** (A) IF images showing H3K27me3 intensity in PLZF^+^ spermatogonia. Yellow arrowheads indicate PLZF^+^ spermatogonia. Dashed lines delineate seminiferous tubules. Scale bars: 10 μm. (B) Quantification of H3K27me3 fluorescent intensity for PLZF^+^ spermatogonia in control and cryptorchid testis. *n*=2 mice per genotype. (C) Representative H3K9me3 signal patterns for PLZF^+^ spermatogonia in control and cryptorchid testes. Cells were classified by the pale and patchy patterns of H3K9me3 signals. Dashed lines delineate seminiferous tubules. Yellow arrowheads indicate PLZF^+^ spermatogonia. Scale bars: 10 μm. (D) Ratio of pale and patchy H3K9me3 patterns in PLZF^+^ spermatogonia. χ^2^ test was used to compare the proportions between the samples (the *P*-value is shown above the plot). *n*=3 mice per genotype. (E) Quantification of pale fluorescent signals for H3K9me3. *n*=3 mice per genotype. (F) Quantification of patchy fluorescent signals for H3K9me3. *n*=3 mice per genotype. (B,E,F) *P*-values (one-tailed Wilcoxon rank sum test) and the number of cells are indicated above the plots. In box plots, data are median (horizontal line), 25th and 75th percentile; whiskers show the range; dots represent outliers (>1.5× the interquartile range).

To investigate whether epigenetic alterations underlie the observed effect of cryptorchidism on spermatogonial maintenance, we performed IF on d30 cryptorchid testes using antibodies for histone modifications. Because reports suggest the importance of repressive histone modifications as temperature-sensitive machinery in various species, we focused our analysis on H3K27me3 and H3K9me3. To compare fluorescent signals in an unbiased manner, we performed each IF experiment treating both control and cryptorchid testicular sections in the same drop of antibody reactions. Our experiments detected little change in the H3K27me3 signal intensity in PLZF^+^ spermatogonia (Fig. 2A,B). When analyzing H3K9me3 signals, we classified the cells into two groups according to the H3K9me3 patterns (Fig. 2C). The first group had pale, faint signals, often with peripheral signals along the nuclear envelope. In the normal spermatogonia, these cells correspond to the undifferentiated population of spermatogonia (Shirakawa et al., 2013). The second group showed patchy foci across the nucleus, which are typically observed in progenitor spermatogonia that are committed to differentiate (Shirakawa et al., 2013). Classification of these cells showed that the ratio of the patchy group increased slightly in the cryptorchid testis, but the difference was not statistically significant (Fig. 2D). Quantification of H3K9me3 fluorescent intensity demonstrated that there is a small increase in the intensity of the patchy cells (*P*=0.04), whereas that of pale cells remained unchanged in cryptorchid testis (*P*=0.19) (Fig. 2E,F). Thus, these results suggest that the effect of cryptorchidism on global H3K27me3 and H3K9me3 modification levels is small.

### Alterations in local histone modifications in cryptorchid spermatogonia

**Fig. 3.**
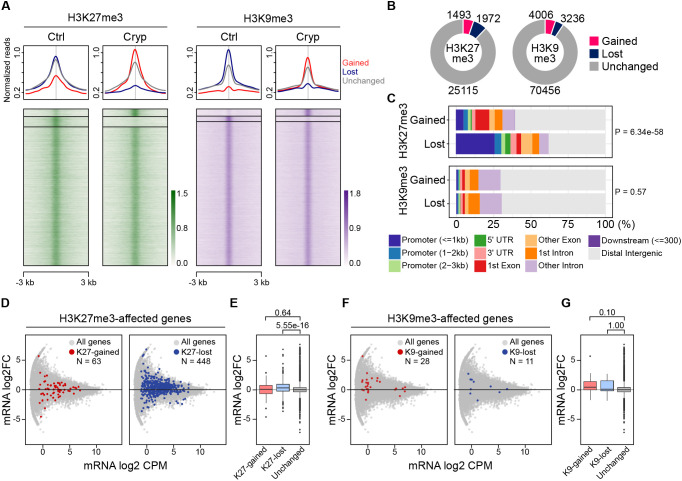
**Altered local histone modifications correlate with gene expression in cryptorchid spermatogonia.** (A) ChIP-seq heatmap showing differential enrichment of H3K27me3 and H3K9me3 peaks in control and cryptorchid GFRa1^+^/KIT^−^ spermatogonia. Merged data were used from triplicate and duplicate samples of H3K27me3 and H3K9me3, respectively. Gained, lost, and unchanged peaks in cryptorchid spermatogonia are shown. Peak centers ±3 kb are displayed. (B) Number of gained and lost ChIP-seq peaks in cryptorchid GFRa1^+^/KIT^−^ spermatogonia. (C) Feature annotation of affected peaks in cryptorchid spermatogonia. *P*-values were calculated by χ^2^ test for the difference in the proportion of promoter peaks (≤1 kb) between the gained and lost fractions. (D) M-A plot showing RNA-seq expression fold change in control and cryptorchid GFRa1^+^/KIT^−^ spermatogonia (*n*=5 control and 7 cryptorchid samples). Genes associated with H3K27me3-gained and lost peaks (within 2 kb of TSSs) are highlighted. (E) Box plot for mRNA levels comparing H3K27me3-gained, -lost, and -unchanged genes. (F) M-A plot for the expression of H3K9me3-associated genes. Affected peaks within 2 kb of TSSs were analyzed. (G) Box plot for the expression of H3K9me3-associated genes. (E,G) *P*-values above the plots were calculated by the one-tailed Wilcoxon rank sum test. In box plots, data are median (horizontal line), 25th and 75th percentile; whiskers show the range; dots represent outliers (>1.5× the interquartile range).

The signals from IF images do not necessarily reflect local changes in histone modifications that may affect mRNA transcription. To investigate histone modification changes at a high resolution, we performed fluorescence-activated cell sorting (FACS) for cryptorchid testes to enrich SSCs. Because d30 cryptorchid samples would exhibit abnormalities derived not only from primary defects but also from various secondary effects, the use of these samples for molecular analyses were not considered suitable. Instead, given the observation that all SSC subgroups complete the cell cycle within a period of 9 days (Nakagawa et al., 2021), we used d10 samples, which would exhibit an early response to the induction of cryptorchidism after the completion of replication-coupled chromatin alterations. We induced cryptorchidism in mice carrying GFRa1-EGFP, which is highly expressed in SSCs (Uesaka et al., 2007). From these mice, we collected undifferentiated spermatogonia (GFRa1-EGFP^+^/KIT^−^) while distinguishing from differentiating spermatogonia (GFRa1-EGFP^+^/KIT^+^) by FACS and analyzed epigenetic changes in SSCs (Fig. S1A-F). Counting of GFRa1^+^/KIT^−^ cells confirmed that the proportion of SSCs relative to all testicular cells was significantly higher in cryptorchid samples than controls (Fig. S1G), reflecting the loss of differentiated germ cells in d10 cryptorchid testes (Fig. 1D). Quantitative reverse transcription PCR (qRT-PCR) analysis using specific primers for marker genes confirmed high enrichment of the collected cells in undifferentiated spermatogonia (Fig. S1H). Using these cells, we performed ChIP-seq for H3K27me3 (*n*=3) and H3K9me3 (*n*=2). Our analysis identified 28,580 H3K27me3 and 77,698 H3K9me3 peaks in GFRa1^+^/KIT^−^ spermatogonia. Of the H3K27me3 peaks, 5.2% (1493 peaks) were gained and 6.9% (1972 peaks) were lost in cryptorchid SSCs (Fig. 3A,B). Similarly, as for H3K9me3, 5.2% (4006 peaks) were gained and 4.2% (3236 peaks) were lost in cryptorchid GFRa1^+^/KIT^−^ spermatogonia (Fig. 3A,B). Consistency of the peaks between replicate datasets was confirmed by Pearson correlation analysis (Fig. S2A,B). Comparison of affected H3K27me3 peaks showed that the lost peaks are more frequently associated with promoters as compared with gained peaks (*P*=6.34e−58) (Fig. 3C). However, there was little difference in the H3K9me3-overlapping features between gained and lost peaks (*P*=0.57) (Fig. 3C). We next performed gene ontology analysis for the genes for which the transcription start sites (TSSs) are located within 2 kb of the affected peaks. Interestingly, although not many terms were found for the H3K27me3-gained genes (*n*=144), H3K27me3-lost genes (*n*=633) were enriched for terms related to development and differentiation (Fig. S2C,D). In contrast, no significant enrichment was found for the H3K9me3 gained (*n*=89) or lost genes (*n*=70). These results suggest that cryptorchidism causes local changes of H3K27me3 and H3K9me3 in SSCs and may affect transcription in different ways.

### Reduced H3K27me3 underlies activation of developmental and proapoptotic genes

To investigate whether altered local histone modifications affect mRNA expression, we performed RNA sequencing (RNA-seq) from d10 cryptorchid spermatogonia (*n*=5 control and 7 cryptorchid samples). Data reproducibility between replicate datasets was confirmed by Pearson correlation analysis (Fig. S2E). Differential expression analysis of GFRa1^+^/KIT^−^ cells identified 446 upregulated and 410 downregulated genes in cryptorchid spermatogonia (Fig. S2F). Gene ontology analysis showed that upregulated genes are associated with regulation of transcription by RNA polymerase II as well as development and differentiation. Similarly, downregulated genes were enriched for development, differentiation and spermatogenesis (Fig. S2G). To investigate whether altered histone modifications affect mRNA expression, we focused on genes for which the TSSs are within 2 kb of the affected ChIP-seq peaks and analyzed their gene expression changes. Strikingly, comparison of control and cryptorchid GFRa1^+^/KIT^−^ spermatogonia indicated an increasing trend of mRNA for the H3K27me3-lost genes, but not for the H3K27me3-gained genes, in cryptorchid spermatogonia (Fig. 3D). Among the H3K27me3-lost and mRNA-affected genes (>1.5-fold changes in mRNA; *n*=234), 170 genes (72.6%) were upregulated. The mRNA increase of H3K27me3-lost genes was significantly greater compared with the H3K27me3-unchanged genes (Fig. 3E). In contrast, only 39 genes were associated with the cryptorchid-affected H3K9me3 peaks with no inverse correlation with mRNA changes compared with the unchanged subset (Fig. 3F,G). Because H3K9me3 is associated with retroelement silencing, we also analyzed expression of short interspersed nuclear elements (SINEs), long interspersed nuclear elements (LINEs), and long terminal repeats (LTRs). This analysis revealed one SINE, one LINE, and 11 LTR subfamilies that were differentially expressed (Fig. S3A-C). However, quantification of H3K9me3 peaks within 2 kb of the LTR copies of the upregulated subfamilies indicated that associated H3K9me3 were not significantly different in cryptorchid SSCs (Fig. S3D). Thus, these results suggested that the H3K27me3 loss is associated with mRNA increase but H3K9me3 changes did not significantly affect gene expression in cryptorchid spermatogonia.

**Fig. 4.**
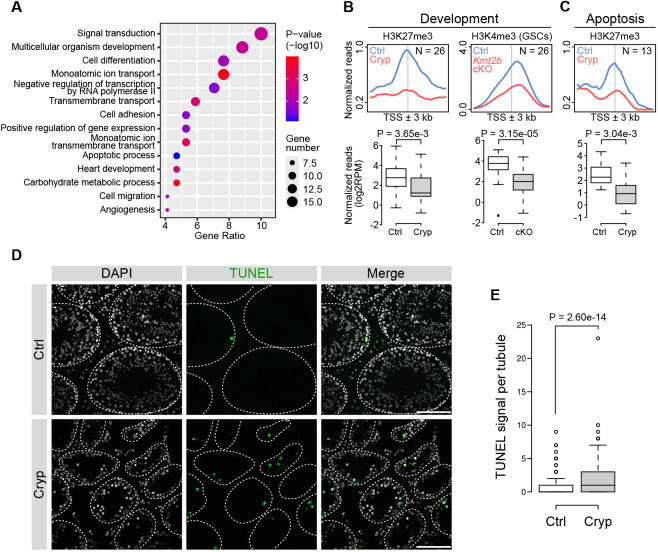
**Activation of developmental and proapoptotic genes in cryptorchid spermatogonia.** (A) Gene ontology for the mRNA-upregulated (>1.5-fold) genes associated with the H3K27me3-lost peaks in GFRa1^+^/KIT^−^ spermatogonia (*n*=170). Background: all genes. Gene ratio: percentage of genes in the GO category over the total number of genes. (B) ChIP-seq peak enrichment over the affected developmental gene TSSs. Developmental genes: enrichment of H3K27me3 at TSSs of the affected genes in control and cryptorchid GFRa1^+^/KIT^−^ spermatogonia (left) and H3K4me3 at the same genes in *Kmt2b* control and the influence of *Kmt2b* cKO in GSCs (right). Values are read count per million mapped reads. Box plots show ChIP-seq read quantification of the corresponding peaks. See Table S3 for the list of the genes analyzed. (C) H3K27me3 enrichment at the affected proapoptotic gene TSSs in GFRa1^+^/KIT^−^ spermatogonia. See Table S3 for the list of the genes analyzed. (D) Representative images showing TUNEL signals in testicular sections. Dashed lines delineate seminiferous tubules. Scale bars: 100 μm. (E) Signal quantification for TUNEL. Number of signals per tube was quantified. *n*=150 tubules each from duplicate samples analyzed. (B,C,E) *P*-values: one-tailed Wilcoxon rank sum test. In box plots, data are median (horizontal line), 25th and 75th percentile; whiskers show the range; dots represent outliers (>1.5× the interquartile range).

The upregulated genes among the H3K27me3-lost genes were enriched for terms related to development and differentiation (‘multicellular organism development’ and ‘cell differentiation’) (Fig. 4A). These categories included developmental genes such as *Dll4*, *Hoxb13*, *Frzb*, and *Kdr* (Fig. S4A). The enrichment of H3K27me3 over the promoters of these genes decreased in cryptorchid GFRa1^+^/KIT^−^ spermatogonia (Fig. 4B). Developmental genes generally carry bivalent chromatin marked by KMT2B-mediated H3K4me3 and PRC2-mediated H3K27me3 (Bernstein et al., 2006). To determine whether the genes affected in cryptorchid spermatogonia exhibit KMT2B-mediated bivalent features in spermatogonia, we analyzed H3K4me3 ChIP-seq data from GSCs in which *Kmt2b* is conditionally knocked out (cKO) by Cre-loxP recombination (Tomizawa et al., 2018). In *Kmt2b* cKO GSCs, the H3K4me3 level at the promoter decreased, suggesting that the cryptorchid-affected genes are the typical KMT2B-targeted bivalent genes in spermatogonia (Fig. 4B, Fig. S4A).

Another interesting category that showed enrichment in the H3K27me3-lost/mRNA-upregulated genes was ‘apoptotic process’ (Fig. 4A), which may contribute to the loss of differentiated germ cells in cryptorchid testes. Consistently, we observed a general increasing trend in well-known apoptosis-related genes such as *Fas*, *Tnfrsf1a*, *Il4*, *Tlr3*, and *Cd2*, with some of the genes showing statistically significant upregulation in cryptorchid GFRa1^+^/KIT^−^ spermatogonia (Fig. S4B). In cryptorchid GFRa1^+^/KIT^−^ spermatogonia, enrichment of H3K27me3 over the affected apoptosis gene promoters decreased (Fig. 4C). To examine whether our cryptorchid testes show an increased event of apoptosis, we performed terminal deoxynucleotidyl transferase (TdT)-mediated d-UTP nick end-labeling (TUNEL) staining. This experiment confirmed a significant increase in the number of apoptotic cells per seminiferous tubules (Fig. 4D,E). Together, these results demonstrate that cryptorchid SSCs have lost a subset of the PRC2-mediated H3K27me3 at KMT2B-mediated bivalent genes as well as at proapoptotic genes, which may lead to premature activation of developmental programs and apoptotic cell death.

### Aberrant activation of KDM6 demethylases in cryptorchid testis

**Fig. 5.**
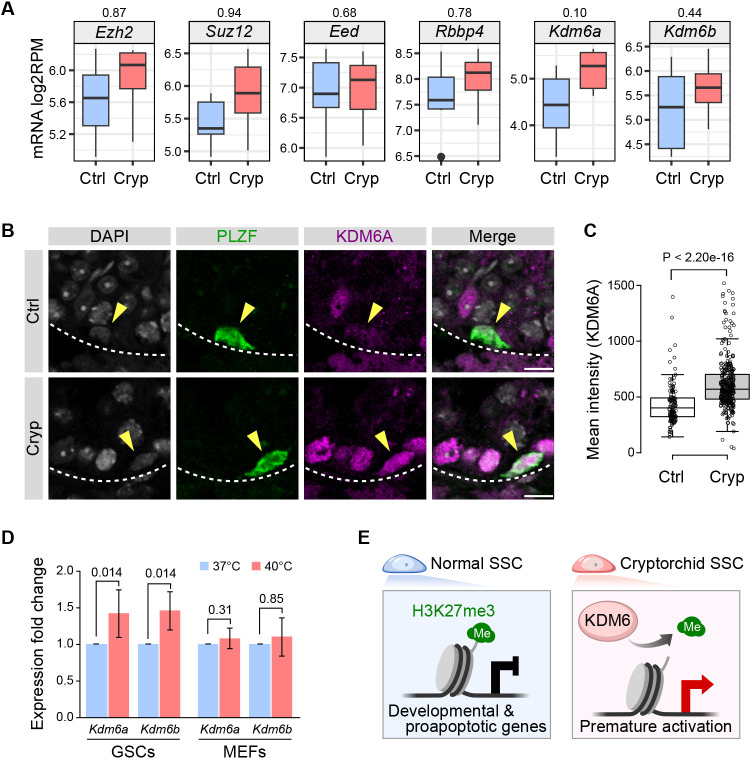
**Increased expression of KDM6 lysine demethylases.** (A) Box plots showing GFRa1^+^/KIT^−^ RNA-seq values for genes involved in deposition or removal of H3K27me3. Data are from *n*=5 control and 7 cryptorchid samples. *P*-values are shown above the plots. (B) Representative IF images from frozen testicular sections for KDM6A. Yellow arrowheads indicate PLZF-expressing spermatogonia. Dashed lines delineate seminiferous tubules. Scale bars: 10 μm. (C) Quantification for KDM6A IF signal intensity in PLZF-expressing spermatogonia. *n*=119 control and 327 cryptorchid cells from duplicate samples analyzed. (D) qRT-PCR showing *Kdm6a* and *Kdm6b* expression in GSCs cultured in different temperatures. Values are mean fold change from the control samples (37°C) from four GSC and three MEF experiments. Error bars represent s.d. (E) Schematic model showing that increased expression of KDM6 proteins (KDM6A/B) leads to H3K27me3 removal and activation of developmental and proapoptotic genes in cryptorchid SSCs that are under heat stress. (A,C,D) *P*-values: one-tailed Wilcoxon rank sum test. In box plots, data are median (horizontal line), 25th and 75th percentile; whiskers show the range; dots represent outliers (A) (>1.5× the interquartile range) and data points (C).

Given the mRNA activation of H3K27me3-lost genes in cryptorchid spermatogonia, we searched for genes responsible for this change. We analyzed the expression of genes involved in deposition or removal of H3K27me3 using the RNA-seq data in GFRa1^+^/KIT^−^ spermatogonia. Major PRC2 genes involved in H3K27me3 deposition (*Ezh2*, *Suz12*, *Eed*, and *Rbbp4*) did not show downregulation in cryptorchid spermatogonia. In contrast, we observed a modest increasing trend of the H3K27 demethylases *Kdm6a* (also known as *Utx*) and *Kdm6b* (also known as *Jmjd3*) in cryptorchid GFRa1^+^/KIT^−^ spermatogonia, although this was not statistically significant (Fig. 5A). This comparison also showed a modest increasing trend of *Ezh2* and *Suz12*, which might be linked to the observed increase of a subset of H3K27me3 peaks (Fig. 3A,B). To validate the upregulation of the H3K27me3 demethylation machinery at the protein level, we performed IF analysis for the KDM6A protein. Consistent with the mRNA changes, d30 cryptorchid testis showed significantly increased fluorescent signals for KDM6A in cryptorchid PLZF^+^ spermatogonia (Fig. 5B,C). Furthermore, IF analysis for the KDM6B protein showed a modest increase of signal intensity in the PLZF^+^ spermatogonia in cryptorchid testis (Fig. S5A,B). These results suggest the involvement of KDM6A and KDM6B in the partial loss of H3K27me3 in cryptorchid SSCs.

### Increased temperature causes *Kdm6a/b* upregulation

Increased temperature is one of the major factors that has been suspected to affect germ cell development in cryptorchid testis (Cobellis et al., 2014; Mieusset and Bujan, 1995). However, it is not easy to assess the effect of temperature on gene expression abnormality *in vivo* while excluding the influence of other external stimuli, such as hormonal changes. To circumvent this issue, we used GSC culture to investigate whether the observed upregulation of *Kdm6a/b* genes in cryptorchid spermatogonia depends on temperature changes. We cultured GSCs under normal (37°C) and heated (40°C) conditions and compared gene expression by qRT-PCR. After 12 h of culture, both *Kdm6a/b* showed a modest but statistically significant upregulation in GSCs cultured under the heated condition (Fig. 5D). In contrast, mouse embryonic fibroblasts (MEFs) cultured in the same condition did not show altered expression of *Kdm6a/b* (Fig. 5D), suggesting that the changes we observed specifically occurred in GSCs. Thus, our results demonstrate that increased temperature induces upregulation of *Kdm6a/b* transcription in spermatogonia.

## DISCUSSION

Although cryptorchidism has been studied since the eighteenth century, the molecular mechanisms responsible for the impaired spermatogenesis remain unclear (Cobellis et al., 2014; Tackett et al., 2007). By performing histological and molecular analyses, we identified partial H3K27me3 alterations in cryptorchid spermatogonia that are correlated with abnormal gene expression. Notably, a subset of H3K27me3-lost genes including developmental and proapoptotic genes showed increased mRNA expression. Our observations resemble premature activation of germline genes in embryonic stem cells with deletion of the H3K9 methyltransferase SETDB1 or PRC1.6 (Mochizuki et al., 2021). These findings suggest a possibility that epigenetic abnormalities may be one of the causes of infertility in cryptorchid children.

In contrast to H3K27me3-dependent genes, changes in H3K9me3 were not inversely correlated with the altered expression of genes or retroelements in cryptorchid spermatogonia. This may be because other regulatory systems, including repressive modifications, compensated for the changes of H3K9me3. Indeed, depletion of the H3K9me3 enzyme SETDB1 in primordial germ cells does not de-repress all H3K9me3-marked genes or endogenous retroviruses, implicating the presence of an alternative repressive pathway (Liu et al., 2014). Instead, we observed that the patchy H3K9me3 showed increased signal intensity in cryptorchid spermatogonia. It is possible that the altered signal patterns reflect changes of long-range chromatin interactions and nuclear organization induced by H3K9 methylation changes in a subset of regions (Padeken et al., 2022). Thus, cryptorchidism may cause alteration of various types of histone modifications and affect gene expression as well as nuclear architecture.

Importantly, we identified an increase of the H3K27 demethylases KDM6A/B in cryptorchid spermatogonia. Our data suggested that KDM6A/B can be increased both at the mRNA and the protein levels in spermatogonia. In wild-type mice, KDM6A is not expressed in undifferentiated spermatogonia, and the conditional loss of KDM6A in the germline does not affect spermatogenesis, although hundreds of genes undergo deregulation (Walters et al., 2023). Germline-specific loss of KDM6B also does not impair spermatogenesis, but the mice sire offspring for a longer period, likely as a result of increased and prolonged maintenance of spermatogonia (Iwamori et al., 2013). Thus, the knockout phenotype from these reports suggests that KDM6A/B are dispensable for spermatogenesis. Conversely, our observation suggests that overexpressed KDM6A/B in SSCs may remove H3K27me3 at genes that should be repressed for normal spermatogenesis. This is consistent with reports that PRC1 and PRC2 are required for SSC self-renewal through H3K27me3 (Hu et al., 2024; Maezawa et al., 2017). Our results thus suggest that partial H3K27me3 changes induced by cryptorchidism associated with gene expression changes may have interfered with normal spermatogonial maintenance.

In GSCs, an increase of culture temperature from 37°C to 40°C was sufficient for the upregulation of *Kdm6a/b* at the mRNA level. This observation resembles a previous report showing that *Kdm6b* is upregulated in a temperature-dependent manner in *Trachemys scripta* (Ge et al., 2018). However, there are some differences: *T. scripta Kdm6b* upregulation occurs at a low temperature of male-producing 26°C and not at the female-producing 32°C; this upregulation takes place in gonadal somatic cells and not in germ cells. At present, the mechanisms leading to temperature-dependent *Kdm6b* upregulation remains unclear, but evidence linking temperature and histone modification changes in different types of cells in animals and plants suggests that a similar phenomenon may be widespread in cells to adapt to environmental changes by altering gene expression (He et al., 2021; Wu et al., 2020). Thus, our data suggest the importance of temperature in maintaining the epigenetic integrity of SSCs.

A limitation of this study is that the induced cryptorchidism in adult mice differs from human congenital cryptorchidism, where the testes have never descended before. Additionally, it is likely that heat-treatment of the GSCs does not accurately reflect the cryptorchid conditions in the testis due to differences in temperature and duration of treatment. Moreover, the changes of KDM6A/B in testicular spermatogonia and GSCs were relatively mild at both the mRNA and protein levels. Further studies are needed to establish a better system to assess the effect of temperature on spermatogenesis through epigenetic changes more precisely and systematically. Nevertheless, this study reveals the temperature-sensitivity of *Kdm6a/b* that may induce epigenetic instability and mRNA dysregulation as a potential cause of spermatogenic abnormalities in cryptorchid testis. These results will aid further studies of cryptorchidism as well as the mechanisms of spermatogenesis.

## MATERIALS AND METHODS

### Mice

Male GFRa1-EGFP (Uesaka et al., 2007) or wild-type ICR (purchased from Japan SLC) mice were kept in a barrier facility at Yokohama City University. They were maintained under standard mouse laboratory conditions in a temperature- and humidity-controlled and light-controlled room on a 12 h light/dark cycle. All mice were allowed to take food and water *ad libitum*. Animal experiments were approved by the Committee for Animal Care and Use at Yokohama City University (approval ID: F-A-20-001, F-A-23-015).

### Genotyping PCR for GFRa1-EGFP mice

Genotyping PCR to identify the mice carrying GFRa1-EGFP was carried out using tail DNA and the primers indicated in Table S1 (Uesaka et al., 2007). Following PCR program was used to amplify the 380 bp fragment: 30 cycles of 94°C for 30 s, 60°C for 30 s, 72°C for 30 s followed by 72°C for 7 min. Amplified DNA fragments were visualized on a 2% agarose gel by electrophoresis.

### GSC culture

GSCs were established and maintained according to previous reports (Kanatsu-Shinohara et al., 2003, 2014). Testes from wild-type ICR mice at postnatal day 7-10 were collected and treated with 0.25% trypsin/EDTA at 37°C for 15 min to prepare a single-cell suspension. After suspending the cells in DMEM/10% fetal calf serum (FCS), the cells were cultured in an incubator at 37°C in 5% CO_2_ on a gelatinized dish supplemented with GS medium. Once the GSC growth stabilized after a few passages, the cells were grown on mitomycin C-inactivated MEFs. For the heat treatment, the cells were split into two dishes that were each placed in 37°C and 40°C incubators simultaneously on day 3 after passage. After 12 h, the cells were trypsinized and centrifuged at 500 ***g*** for 5 min. The cells were further suspended in GS medium and replated on gelatinized dishes for 30 min at the same temperature, and unadhered cells were collected for experiments. The MEFs were cultured in the same way to use for control experiments. The cells were checked for *Mycoplasma* contamination using the MycoStrip Mycoplasma Detection Kit (InvivoGen, rep-mys-10).

### Cryptorchid surgery

Unilateral cryptorchidism was surgically induced in 8- to 10-week-old male mice according to the method reported previously (Zheng et al., 2019). Briefly, mice were anesthetized, and the left testis was pulled up and attached to the abdominal wall, while the right testis was left in the scrotum as control (Fig. 1A). For the histological analysis, d30 post-surgery testes were used, whereas d10 post-surgery testes were used for RNA-seq and ChIP-seq analyses.

### H&E staining

For the H&E staining, testes were fixed in Bouin's solution at 4°C overnight. The samples were transferred to 70% ethanol, then immersed in xylene, and embedded in paraffin. The sliced sections (8 µm thickness) were used for H&E staining by standard procedures. A Keyence BZ-X800 microscope was used for microscopy analysis.

### IF analysis

Histological experiments were performed as previously reported (Ohmura et al., 2004; Shirakawa et al., 2013; Tomizawa et al., 2024). Testes were fixed in 4% paraformaldehyde for either 5 h (control) or 2.5 h (cryptorchid) at 4°C. Tissues were transferred to increasing concentrations (10-30%) of sucrose in PBS at 4°C, and embedded in Tissue-Tek O.C.T compound (Sakura Finetek). Sliced sections were treated with blocking solution (2% bovine serum albumin) for 30 min at room temperature, and incubated with primary antibody at 4°C overnight. After washing with PBS, the samples were treated with secondary antibody for 1 h at room temperature. Following DAPI staining, the sections were mounted with ProLong Glass (Invitrogen) and observed using a confocal laser scanning microscope (Olympus FV-1000 or Nikon AX/AXR). Control and cryptorchid sections were treated together in the same drop of reactions to avoid biased staining. The list of the antibodies used for IF are provided in Table S2. The signal intensity was quantified using NIH ImageJ software.

### Apoptosis assay

TUNEL staining was performed on frozen sections using the ApopTag Plus Fluorescein in Situ Apoptosis Detection Kit (Chemicon International, S7111) according to the manufacturer's protocol. After DAPI was applied, the samples were mounted with ProLong Gold (Invitrogen) and observed using an Olympus FV-1000 microscope. TUNEL-positive cells were quantified by counting the number of signals >10 µm^2^.

### Isolation of spermatogonia

Seminiferous tubules from adult GFRa1-EGFP mice (8-10 weeks old) were dissociated in cold PBS to remove interstitial cells. The tissues from each testis were digested in 1 ml of PBS containing 1 mg of collagenase (Sigma-Aldrich, C0130) and 1 unit of RQ1 DNase (Promega, M6101) for 9 min at either 32°C (control) or 37°C (cryptorchid). Next, 10 ml of ice-cold PBS containing 5% FCS was added to stop the reaction. The cells were filtrated using a 100 µm strainer, and pelleted by centrifugation at 400 ***g*** for 5 min at 4°C. The pellet was resuspended in 500 µl of 5% FCS in PBS and reacted with 10 µl of APC-conjugated anti-KIT antibody (BioLegend, 105812) for 30 min at 4°C. The cells were then washed three times in 5% FCS in PBS and labeled with 2 µg/ml of propidium iodide (Sigma-Aldrich) to identify dead cells. After the removal of multicell droplets and dead cells, GFRa1^+^/KIT^−^ cells were identified and sorted using SONY MA900. The sorted cells were analyzed by qRT-PCR to check the purity of spermatogonia.

### qRT-PCR

Total RNA was purified from the sorted spermatogonia (∼4000 cells), GSCs (∼1.0×10^5^ cells), or MEFs (∼1.0×10^5^ cells) using Isogen (Nippon Gene) and treated with RQ1 DNase (Promega) at 37°C for 30 min. The reaction was stopped by adding Stop Solution and incubating for 10 min at 65°C. First-strand cDNA was synthesized from <400 ng of total RNA using Superscript IV (Invitrogen, 18090010) according to the manufacturer's instructions. The cDNA was diluted 1:20 and used for qPCR with FastStart SYBR Green Master (Roche, 4673484001) on Bio-Rad CFX96. Cycle threshold (Ct) values for each gene were normalized for the mean Ct values of *Gapdh* and *Hprt1* using a previously published method (Pfaffl, 2001). The primers used are listed in Table S1.

### ChIP-seq

ChIP-seq for histone modifications was performed using 1000-2000 sorted spermatogonia according to the carrier DNA-assisted ChIP-seq (CATCH-seq) for low-input experiments reported previously (Matsuwaka et al., 2024; Zhu et al., 2021). Carrier DNA oligos digestible with I-SceI (NEB) were used to minimize the loss of the ChIP DNA (purchased from IDT; Table S1). The antibodies used for the ChIP reaction are listed in Table S2. Libraries were generated from the purified ChIP DNA using the NEBNext Ultra II DNA Library Prep Kit for Illumina (NEB, E7645S). The libraries were indexed with NEBNext Multiplex Oligos for Illumina (NEB, E7600S) and pooled for sequencing with Illumina HiSeq X or NovaSeq X Plus, with a paired-end mode for 150 bp.

### RNA-seq

Total RNA was isolated from ∼1000 spermatogonia using Isogen and cDNA was prepared from one-fifth of the RNA obtained using the Smart-seq2 method (Picelli et al., 2013). The purified cDNA was used for library generation using Nextera XT DNA Sample Preparation Kit (Illumina, FC-131-1096) and Nextera XT Index Kit (Illumina, FC-131-1001) according to manufacturer's instructions. Completed libraries were pooled, quantitated with Agilent TapeStation 4150, and sequenced using Illumina HiSeq X or NovaSeq X Plus, with a paired-end mode for 150 bp.

### Data analysis

ChIP-seq reads were quality-checked (FastQC; Babraham Bioinformatics), adapter- and quality-trimmed (Trim Galore! version 0.6.6; Babraham Bioinformatics), and mapped onto the mouse genome (mm10) using Bowtie 2 version 2.5.1 with the parameter -N 1. Mapped reads were quality filtered (>10) and duplicates were removed by Picard MarkDuplicates version 2.26.0 and sorted bam files were produced by SAMtools (Danecek et al., 2021). ChIP-seq peak identification was performed using the MACS2 function (Zhang et al., 2008) of SeqMonk, analyzing ChIP replicates over the input sample with the default parameter (cutoff: 1.0e−5), and the peaks showing significant difference between control and cryptorchid samples (gained and lost peaks) were identified by edgeR version 4.2.2 (Robinson et al., 2010) (>1.5-fold difference; *P*<0.05) after filtering for low-read count per peak [log2 reads per million (RPM)>1 in at least one dataset]. For the final analysis and visualization of the data, de-duplicated bam files from respective replicate samples were merged by SAMtools and treated as either control or cryptorchid data. The ngs.plot software version 2.47 (Shen et al., 2014) was used to visualize ChIP-seq peak enrichment. Peak annotation for genomic features was performed using ChIPseeker version 1.40.0 (Yu et al., 2015). Genes for which TSSs are within 2 kb of the cryptorchid-affected ChIP-seq peaks were used for downstream gene expression analysis after filtering for low expression [log2 count per million read (cpm) calculation by edgeR>−1].

For gene expression analysis, raw RNA-seq reads were adapter- and quality-trimmed (Trim Galore!) and mapped onto the mouse genome (mm10) using HISAT2 version 2.1.0 (Kim et al., 2015). Read count was performed using SeqMonk version 1.48.1 (Babraham Bioinformatics) for the protein-coding gene annotation from the UCSC Table Browser. edgeR software was used to identify differentially expressed genes between control and cryptorchid spermatogonia (>2-fold difference; *P*<0.05 unless otherwise indicated). For the final analysis and visualization of the data, mapped bam files from replicate samples were merged by SAMtools and treated as either control or cryptorchid data.

Pearson correlation analysis for replicate datasets was performed using top 1000 differentially expressed genes (RNA-seq) or differentially enriched MACS2 peaks (ChIP-seq) between control and cryptorchid samples. Gene ontology analysis was performed with DAVID (Huang et al., 2009a,b) using all genes as background.

For the expression analysis of retroelements, RNA-seq reads were counted using the RepeatMasker annotation obtained from the UCSC Table Browser. Total read count for each repName was calculated and significant difference was estimated using edgeR (>2-fold difference; *P*<0.05). H3K9me3 ChIP-seq peaks within 2 kb of elements were analyzed for comparison between control and cryptorchid samples for differential enrichment.

### Statistical analysis

Number of samples and statistical methods used are indicated in figure legends. RNA-seq datasets having excessive numbers of duplicates and ChIP-seq datasets showing poor signal-to-noise ratio were excluded from analysis. No randomization was used, and investigators were aware of the allocation of sample groups during analysis.

## Supplementary Material

10.1242/develop.204239_sup1 Supplementary information
