# Supplementary material for: Abnormal H3K27me3 underlies degenerative spermatogonial stem cells in cryptorchid testis
Source: Development. 2025 Jan 16;152(2):dev204239. doi: 10.1242/dev.204239 (PMC11829757; doi:10.1242/dev.204239)
Supplement: Supplementary information [file develop-152-204239-s1.pdf]

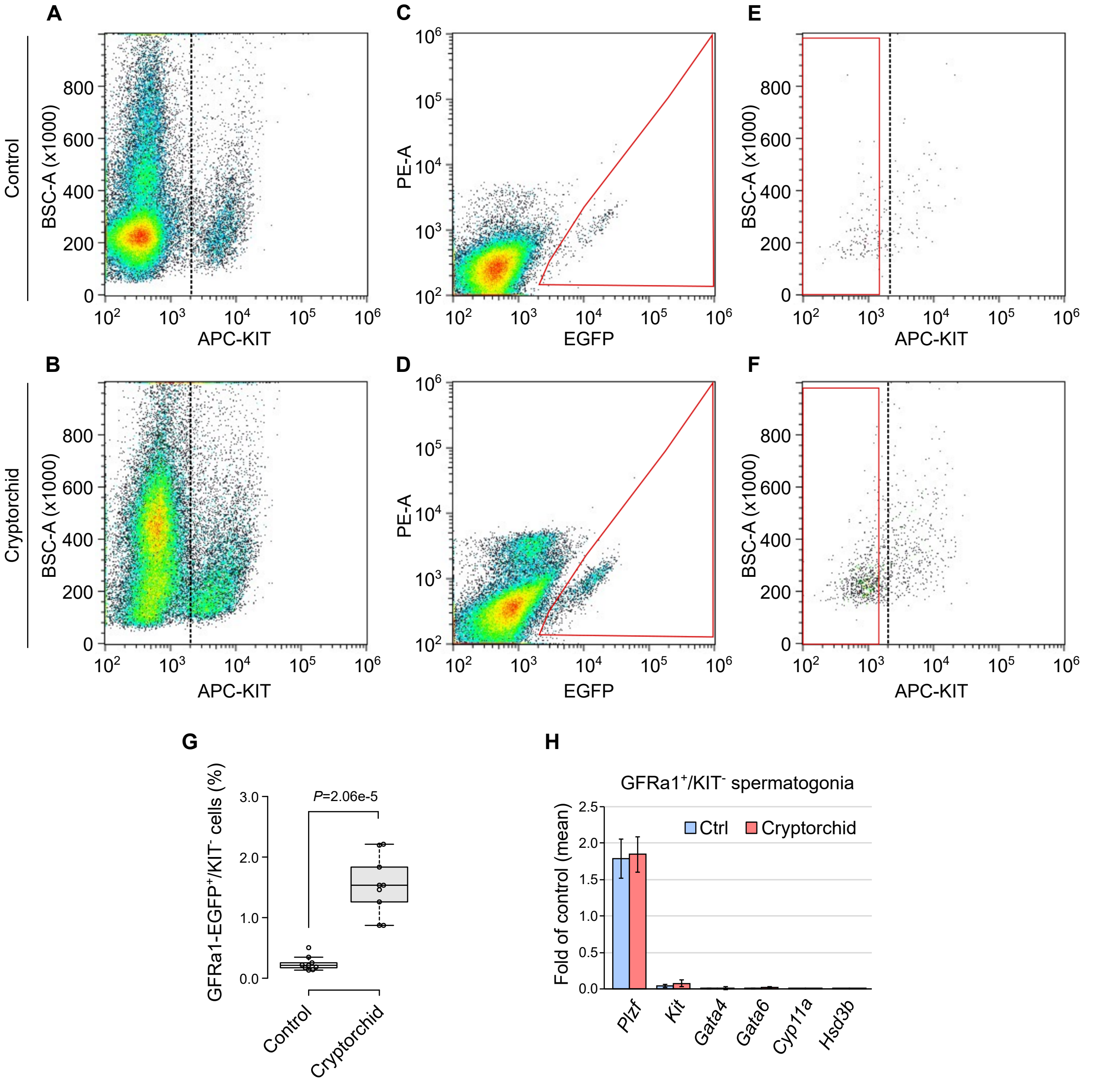

Fig. S1. Isolation and analysis of undifferentiated spermatogonia.

(A, B) FACS plots for control and cryptorchid samples for the identification of KIT$^-$ and KIT$^+$ fractions using all testicular cells. The plots were generated after the removal of multicell droplets and dead cells using propidium iodide (PI).

(C, D) Selection of GFRa1-EGFP spermatogonia (red triangles) in the control **(C)** and cryptorchid **(D)** testicular cell suspensions.

(E, F) The gated EGFP$^+$ areas (red triangles) from **(C)** and **(D)** were further analyzed for the identification of KIT$^-$ and KIT$^+$ fractions according to the plots in **(A)** and **(B)**, and KIT$^-$ spermatogonia were collected (red rectangles).

(G) Percentage of GFRa1-EGFP$^+$/KIT$^-$ cells among the total testicular cell suspension as calculated from the FACS cell count. Data are from 8 control and 9 cryptorchid experiments. Box plot: data are median (horizontal line), 25th and 75th percentile; whiskers show the range; dots represent data points. *P*-values were calculated by one-tailed Wilcoxon rank sum test.

(H) qRT-PCR for testicular marker genes in GFRa1$^+$/KIT$^-$ spermatogonia (*n*=3 per genotype). *Plzf*: undifferentiated spermatogonia; *Kit*: differentiating spermatogonia; *Gata4* and *Gata6*: Sertoli cells; *Cyp11a* and *Hsd3b*: Leydig cells. The values of each gene were normalized for the expression of the mean values for *Gapdh* and *Hprt1*. Error bars represent s.d.

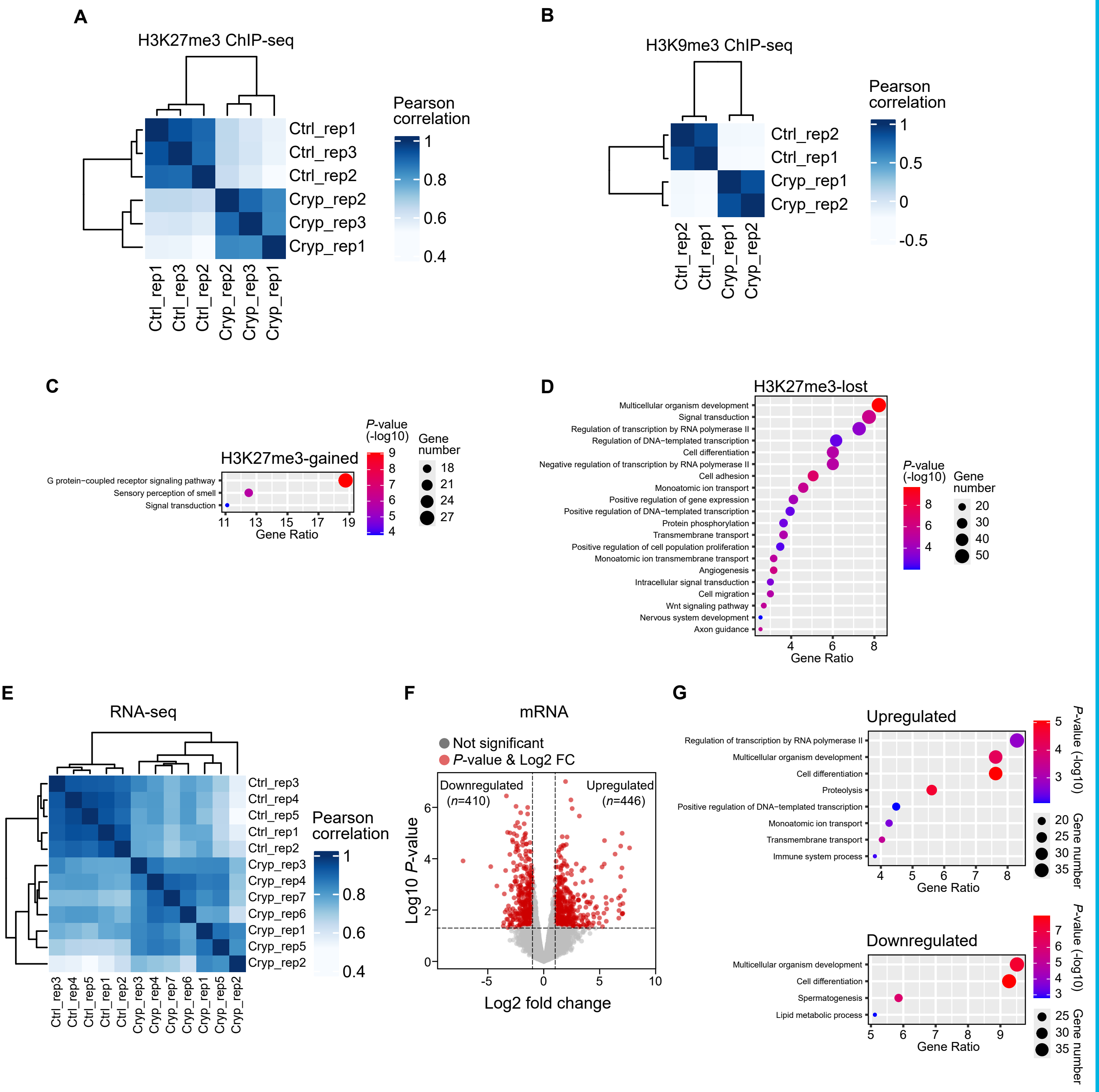

Fig. S2. Altered local H3K9me3 in cryptorchid spermatogonia

(A) Heatmap of the Pearson correlation matrix between replicate datasets for H3K27me3 ChIP-seq. Top 1000 differential MACS2 peaks were used for analysis.

(B) Pearson correlation matrix for the H3K9me3 ChIP-seq replicates using top 1000 differential MACS2 peaks.

(C) Gene ontology enrichment (biological process) for the genes associated with the cryptorchid-affected H3K27me3-gained peaks (< 2 kb of TSS; *n*=144) in GFRa1$^+$/KIT$^-$ spermatogonia.

(D) Gene ontology enrichment (biological process) for the genes associated with the cryptorchid-affected H3K27me3-lost peaks (< 2 kb of TSS; *n*=633).

(E) Pearson correlation matrix for the RNA-seq replicate datasets from GFRa1$^+$/KIT$^-$ spermatogonia using top 1000 differentially expressed genes.

(F) Volcano plot showing differentially expressed genes between control and cryptorchid spermatogonia (GFRa1$^+$/KIT$^-$).

(G) Gene ontology enrichment (biological process) for the RNA up- and down-regulated genes in cryptorchid GFRa1$^+$/KIT$^-$ spermatogonia (*n*=446 and 410, respectively).

(C, D, G) Gene ratio: percentage of genes in the GO category over the total number of genes. ChIP-seq: *P*-value < 0.1; gene number > 7 are shown. RNA-seq: *P*-value < 0.01; gene number > 15 are shown.

Development • Supplementary information

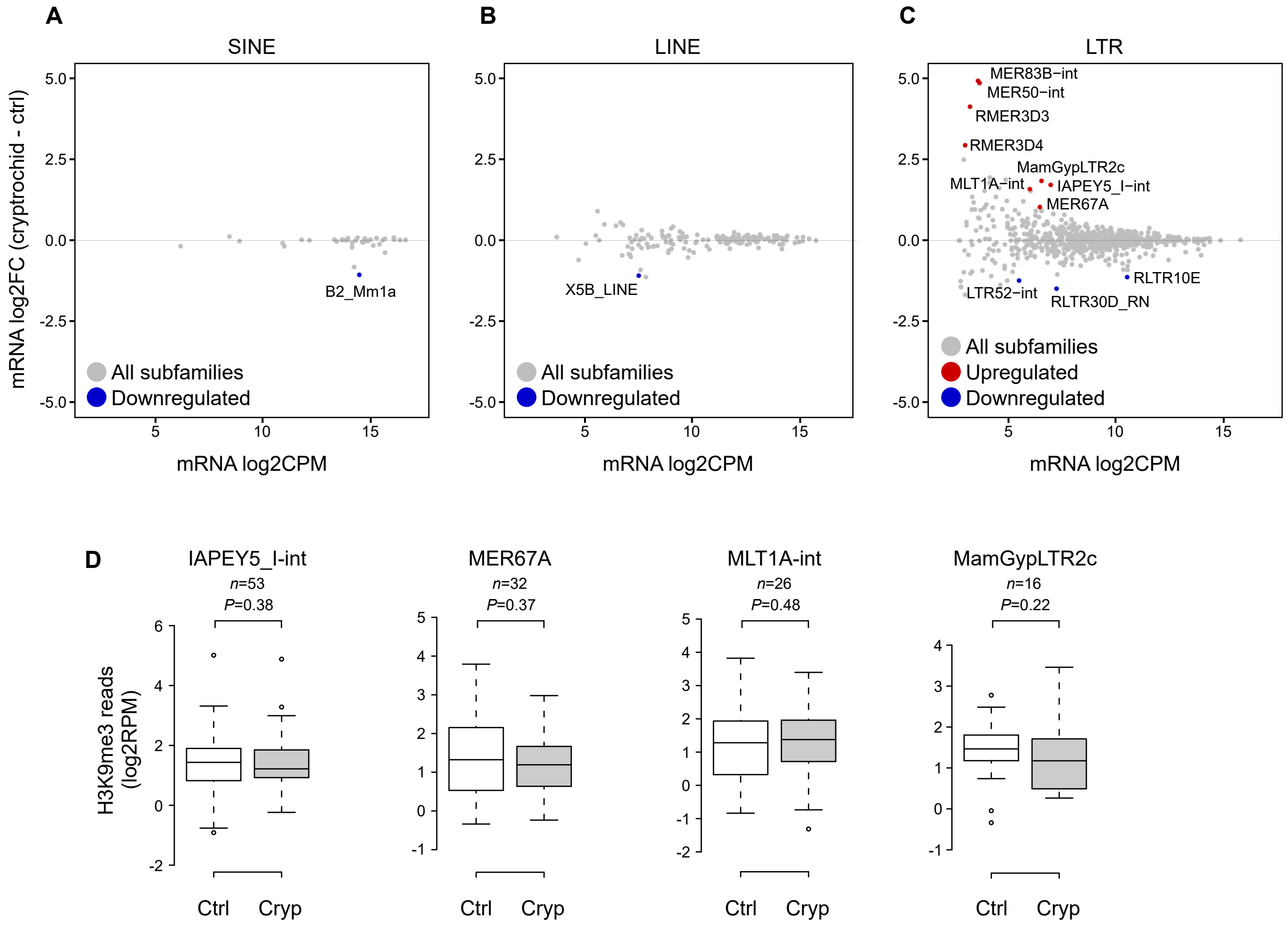

Fig. S3. Expression of retroelements in cryptorchid spermatogonia.

(A) M-A plot showing expression changes of SINE subfamilies in GFRa1^{+}/KIT^{-} spermatogonia.

(B) Expression of LINE subfamilies in GFRa1^{+}/KIT^{-} spermatogonia.

(C) Expression of LTR subfamilies in GFRa1^{+}/KIT^{-} spermatogonia.

(D) Box plots showing H3K9me3 reads at MACS2 peaks within 2 kb of upregulated LTR elements. Only the elements having multiple copies associated with affected H3K9me3 peaks are shown. Data are median (horizontal line), 25th and 75th percentile; whiskers show the range; dots represent outliers (>1.5 x the interquartile range). The number of copies analyzed are shown above the plots. *P*-values above the plots were calculated by one-tailed Wilcoxon rank sum test.

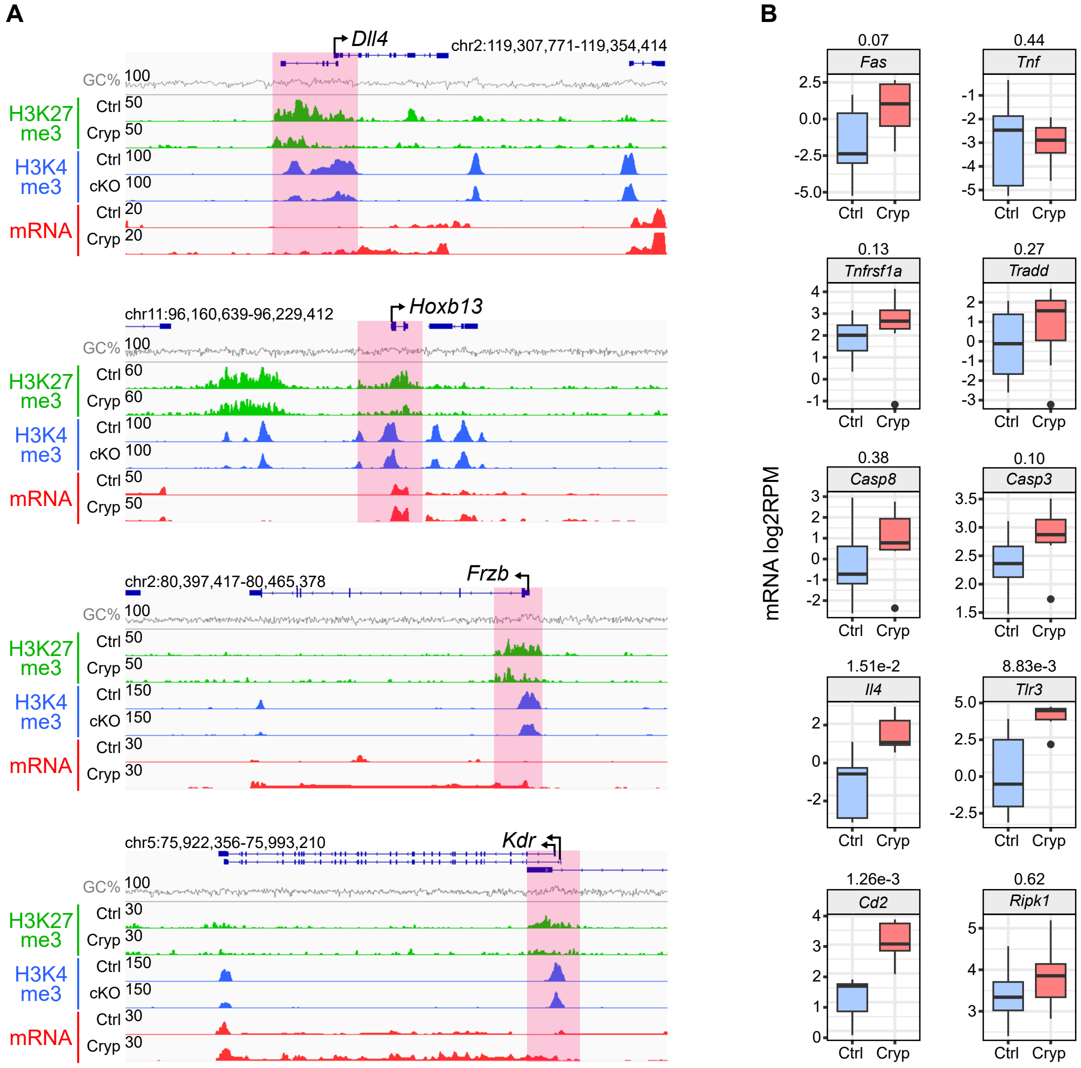

Fig. S4. Activation of the proapoptotic and developmental genes in cryptorchid spermatogonia

(A) Snapshots showing changes in H3K27me3 and mRNA for the representative developmental genes in GFRa1^{+}/KIT^{-} spermatogonia. Affected H3K27me3 regions overlapping promoters are highlighted. H3K4me3 ChIP-seq tracks from *Kmt2b* control and cKO GSCs are included to show bivalency and dependency on KMT2B at the affected regions.

(B) Box plots showing RNA-seq expression changes of genes involved in apoptosis in cryptorchid GFRa1^{+}/KIT^{-} spermatogonia (*n*=5 control and 7 cryptorchid samples). *P*-values above the plots were calculated by one-sided Wilcoxon rank sum test. Box plots: data are median (horizontal line), 25th and 75th percentile; whiskers show the range; dots represent outliers (>1.5 x the interquartile range).

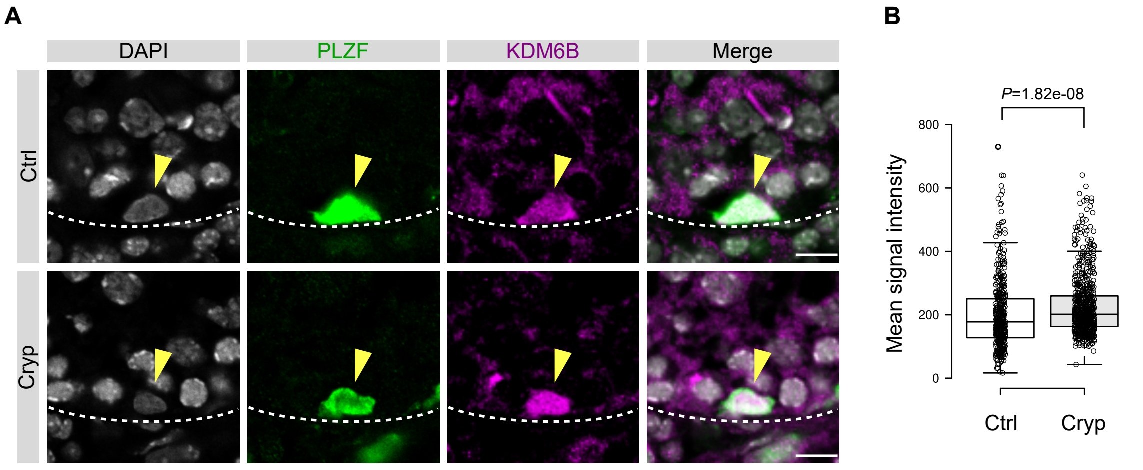

Fig. S5. KDM6B expression in cryptorchid spermatogonia.

(A) Representative IF images from frozen testicular sections showing KDM6B expression in spermatogonia. Yellow arrowheads indicate PLZF$^+$ spermatogonia. Dotted lines delineate seminiferous tubules. Scale bars: 10 µm.

(B) KDM6B signal quantification in PLZF$^+$ spermatogonia. Control (n=390) and cryptorchid (n=631) cells from duplicate samples quantified. P-value: one-tailed Wilcoxon rank sum test. Data are median (horizontal line), 25th and 75th percentile; whiskers show the range; dots represent data points.

Table S1. Oligonucleotides used for genotyping PCR, qRT-PCR, and ChIP-seq.

Name	Forward/reverse	Sequence	Purpose
GFRa1 F	Fw	CTTCCAGGTTGGGTCGGAACTGAACCC	Genotyping
GFRa1 R	Rv	TTTACGTCGCCGTCCAGCTCGA	Genotyping
Plzf F	Fw	AACGGTTCCTGGACAGTTTG	qRT-PCR
Plzf R	Rv	CCATGTCCGTGCCAGTATG	qRT-PCR
Kit F	Fw	CGCCTGCCGAAATGTATG	qRT-PCR
Kit R	Rv	GGGTTGCAGTTTGCCAAG	qRT-PCR
Gata4 F	Fw	CGAGGGTGAGCCTGTATGTA	qRT-PCR
Gata4 R	Rv	ACCTGCTGGCGTCTTAGATT	qRT-PCR
Gata6 F	Fw	GAGCTGGTGCTACCAAGAGG	qRT-PCR
Gata6 R	Rv	TGCAAAAGCCCATCTCTTCT	qRT-PCR
Cyp11a F	Fw	AAGTATGGCCCCATTTACAGG	qRT-PCR
Cyp11a R	Rv	GAGTATCGACGCATCCTTGG	qRT-PCR
Hsd3b F	Fw	GCTCCAGACTGGGACTGCTGACAC	qRT-PCR
Hsd3b R	Rv	AATCCTCTGGCCCAAAAACCCTC	qRT-PCR
Gapdh F	Fw	CAATGTGTCCGTCGTGGATCT	qRT-PCR
Gapdh R	Rv	GCCTGCTTCACCACCTTCTT	qRT-PCR
Hprt1 F	Fw	GCCCCAAAATGGTTAAGGTT	qRT-PCR
Hprt1 R	Rv	CAAGGGCATATCCAACAACA	qRT-PCR
Kdm1a F	Fw	TCTGGAAAATTTTGTGGTGCT	qRT-PCR
Kdm1a R	Rv	AGATGAGGCGGATGGTAATG	qRT-PCR
Kdm1b F	Fw	CTACAGGGCGTGGTGGTG	qRT-PCR
Kdm1b R	Rv	CCTCATCGAGACGTGCTG	qRT-PCR
Carrier oligo	Fw	/5AmMC6/GTAGGGATAACAGGGTAATTA<br>GGGATAACAGGGTAATTAGGGATAACAG<br>GGTAATTAGGGATAACAGGGTAATTAGG<br>GATAACAGGGTAATTAGGGATAACAGGG<br>TAAT*/3AmMO/	ChIP-seq
Carrier oligo	Rv	/5AmMC6/GATTACCCTGTTATCCCTAATTA<br>CCCTGTTATCCCTAATTACCCTGTTATCCC<br>TAATTACCCTGTTATCCCTAATTACCCTGT<br>TATCCCTAATTACCCTGTTATCCC<br>TA*/3AmMO/	ChIP-seq

Development • Supplementary information

Table S2. Antibodies used in this study.

Name	Host	Dilution/ amount	Source	Cat#	RRID	Batch#
PLZF	Goat	1:200 (IF)	R&D Systems	AF2944	AB_2218943	VUG0212101R
H3K27me3	Rabbit	1:800 (IF)<br>0.25 µg (ChIP)	Millipore	07-449	AB_310624	2382150
H3K9me3	Rabbit	1:200 (IF)<br>0.25 µg (ChIP)	Abcam	ab8898	AB_306848	GR306402-1
KDM6A/UTX	Rabbit	1:300 (IF)	Cell Signaling	33510	AB_2721244	3, Ref. 12/2019
KDM6B/JMJD3	Rabbit	1:200 (IF)	Cell Signaling	3457	AB_1549620	2, Ref. 05/2017
Alexa Plus 647 anti-goat	Donkey	1:200 (IF)	Invitrogen	A32849	AB_2762840	UD282514
Alexa 488 anti-rabbit	Donkey	1:200 (IF)	Molecular Probes	A21206	AB_2535792	2072687

Table S3. List of the genes associated with developmental or apoptotic terms.

Gene	Chr	Start	End	Strand	ID	Related_term
Arhgef28	13	97899469	98206439	-	ENSMUSG00000021662	Development
Bmp6	13	38345107	38500302	+	ENSMUSG00000039004	Development
Dll4	2	119325784	119335962	+	ENSMUSG00000027314	Development
Ebf4	2	130295169	130370481	+	ENSMUSG00000053552	Development
Ednra	8	77663031	77724464	-	ENSMUSG00000031616	Development
Ephb2	4	136647539	136835988	-	ENSMUSG00000028664	Development
Fat3	9	15910189	16501285	-	ENSMUSG00000074505	Development
Foxc1	13	31806633	31812476	+	ENSMUSG00000050295	Development
Frzb	2	80411970	80447625	-	ENSMUSG00000027004	Development
Gas7	11	67455091	67688990	+	ENSMUSG00000033066	Development
Hoxc13	15	102921103	102928814	+	ENSMUSG00000001655	Development
Hspb7	4	141420779	141425311	+	ENSMUSG00000006221	Development
Kdr	5	75932827	75978458	-	ENSMUSG00000062960	Development
Lta	17	35203165	35205351	-	ENSMUSG00000024402	Development
Ltb	17	35194439	35196320	+	ENSMUSG00000024399	Development
Ltbr	6	125306571	125313885	-	ENSMUSG00000030339	Development
Mixl1	1	180693043	180697034	-	ENSMUSG00000026497	Development
Nfkb2	19	46304320	46312385	+	ENSMUSG00000025225	Development
Ppara	15	85734983	85802819	+	ENSMUSG00000022383	Development
Ryr2	13	11553102	12106945	-	ENSMUSG00000021313	Development
Sema3c	5	17574281	17730268	+	ENSMUSG00000028780	Development
Slfn5	11	82951349	82964840	+	ENSMUSG00000054404	Development
Sox8	17	25565892	25570686	-	ENSMUSG00000024176	Development
Tll2	19	41082753	41206835	-	ENSMUSG00000025013	Development
Tnni3	7	4518305	4524229	-	ENSMUSG00000035458	Development
Vegfb	19	6982473	6987651	-	ENSMUSG00000024962	Development
C1ql3	2	13003457	13011806	-	ENSMUSG00000049630	Apoptosis
Cbln4	2	172036233	172043466	-	ENSMUSG00000067578	Apoptosis
Col8a2	4	126286793	126314330	+	ENSMUSG00000056174	Apoptosis
Emp2	16	10281749	10313968	-	ENSMUSG00000022505	Apoptosis
Kcnip3	2	127456498	127522094	-	ENSMUSG00000079056	Apoptosis
Lta	17	35203165	35205351	-	ENSMUSG00000024402	Apoptosis
Ltb	17	35194439	35196320	+	ENSMUSG00000024399	Apoptosis
Ltbr	6	125306571	125313885	-	ENSMUSG00000030339	Apoptosis
Prkcb	7	122288751	122634402	+	ENSMUSG00000052889	Apoptosis
Sulf1	1	12692277	12861192	+	ENSMUSG00000016918	Apoptosis
Tmem102	11	69803603	69805624	-	ENSMUSG00000089876	Apoptosis
Tnfrsf21	17	43016555	43089189	+	ENSMUSG00000023915	Apoptosis
Traf1	2	34941750	34961772	-	ENSMUSG00000026875	Apoptosis
